# Sleep Disturbance as a Predictor of Learning and Memory in Older Adults: The Moderating Role of Depression

**DOI:** 10.1093/geroni/igaf122.3103

**Published:** 2025-12-31

**Authors:** HyeRim Ryu, Cari Cohen, Jerry J Sweet, Christopher Williams, Elizabeth K Geary, Julia Thomas, Leslie Guidotti Breting

**Affiliations:** Rosalind Franklin University of Medicine and Science, Glenview, Illinois, United States; Endeavor Health - NorthShore Hospitals, Chicago, Illinois, United States; Endeavor Health - NorthShore Hospitals, Chicago, Illinois, United States; Endeavor Health - NorthShore Hospitals, Chicago, Illinois, United States; Endeavor Health - NorthShore Hospitals, Chicago, Illinois, United States; Endeavor Health - NorthShore Hospitals, Chicago, Illinois, United States; Endeavor Health - NorthShore Hospitals, Chicago, Illinois, United States

## Abstract

Sleep disturbance, a risk factor for depression (Steiger & Pawlowski, 2019) and memory decline (Crowley, 2011), has become increasingly prevalent among older adults (Reynolds & Adams, 2019). Depressive symptoms are widely associated with lower cognitive functioning in this population (Wei et al., 2019). This study thus investigated whether depressive symptoms moderated the association between sleep disturbance and memory performance. Participants were older adults who received neuropsychological evaluations in an outpatient setting (N = 726, Mage = 76.08, SDage = 6.60). Auditory and visual memory were assessed using the Hopkins Verbal Learning Test (HVLT) and the Brief Visual Memory Test (BVMT), respectively. Depression was assessed using the Geriatric Depression Scale (GDS). The presence or absence of sleep disturbance was assessed during the clinical interview. Results demonstrated that sleep disturbance predicted HVLT immediate recall (p < .01), delayed recall (p = .03), percent retained (p = .01), and recognition discrimination scores (p = .04). GDS scores only moderated the relationship between sleep disturbance and immediate recall scores, such that the negative effect of sleep disturbance on immediate recall became stronger as depressive symptoms increased (p < .01). Regarding visual memory performances, GDS scores moderated the relationship between sleep disturbance and BVMT immediate recall (p = .04), delayed recall (p = .02), and percent retained (p = .01), with particularly strong moderation for delayed recall scores (R² Change = .14). These findings further underscore the complex interplay between comorbid sleep disturbance and depressive symptoms impacting learning and memory among older adults.

